# The Importance of Physical Activity in Preventing Fatigue and Burnout in Healthcare Workers

**DOI:** 10.3390/healthcare11131915

**Published:** 2023-07-03

**Authors:** Ildikó Balatoni, Henrietta Varga Szépné, Tímea Kiss, Umar Gambo Adamu, Adam Michał Szulc, László Csernoch

**Affiliations:** 1 Clinical Center, University of Debrecen, 4032 Debrecen, Hungary; hvarga@med.unideb.hu (H.V.S.); kiss.timea@med.unideb.hu (T.K.); 2 Ihrig Károly Doctoral School of Management and Business, University of Debrecen, 4032 Debrecen, Hungary; umar.gambo@med.unideb.hu; 3 Faculty of Health Sciences and Physical Education, Kazimierz Wielki University, 85-064 Bydgoszcz, Poland; aszul@ukw.edu.pl; 4 Department of Physiology, Faculty of Medicine, University of Debrecen, 4032 Debrecen, Hungary; csl@edu.unideb.hu

**Keywords:** health industry, healthcare workers, sports habits, healthy lifestyle, physical activity

## Abstract

The workers of the health sector are important to the country’s economy in many ways. Healthy and rested workers are highly valuable to the public health sector and give a good perception of their work to patients and society. It is thus important to have a sufficient number of healthy working staff in healthcare institutions who do not have work fatigue and burnout. A total of 987 employees—doctors, professional staff, and others—of a large healthcare institution in Hungary voluntarily participated in a survey regarding their lifestyle and physical activity habits and answered the questions anonymously. Women reported less leisure time (*p* < 0.02), with 54.9% of female respondents saying that they did not exercise regularly, and fatigue was more common among them (*p* < 0.001). In this respect, the healthcare workers’ responses did not differ from those of the overall population. The most common sports were cycling (17.7%), running (15.4%), and working out in a gym (12.3%). Reasons for not participating in sports included lack of time (70.2%) and fatigue (43.9%) as the most frequent responses. Healthcare workers are exposed to a number of risks that require particular attention to maintain their health. Employers should thus focus on implementing programs that prevent burnout and promote healthy lifestyles.

## 1. Introduction

Regular physical activity plays a positive role in maintaining physical fitness, health, and mental well-being, and in preventing depression. As early as the 1990s, researchers pointed to the beneficial effects of regular exercise in the treatment of stress, anxiety, and depression [1,2,3]. The relation of physical activity to health and mental well-being has since been a topic of continuous discussion, and the area is well researched with up-to-date findings that would continuously help people to stay healthy and have mental stability. A recent study assessed the importance of maintaining a healthy lifestyle such as participating in physical activity, eating a healthy diet, etc., in reducing obesity and improving health conditions [4]. Individuals who maintain regular physical activity are found to have sustained happiness, self-esteem, and optimism as compared to those who do not exercise regularly [5]. Physical activity thus helps to build confidence, is seen as therapeutic intervention, and can certainly improve one’s health and well-being [6].

Another study emphasized the importance of a nature prescription, which includes regular physical activity, in improving human health including blood pressure, depression, and anxiety [7]. An intervention in exercise-related activities improves the health of employees [8]. Studies suggested that individuals of low socio-economic upbringing are less likely to engage in physical activity [9,10]. High-intensity physical activity improves one’s cardiorespiratory fitness; however, in their study, the authors of [11] found insufficient intensity in the type of physical activity recommended by WHO. It is also reported that one’s mental well-being is hugely associated with their physical activity level. Engaging in physical activity improves the mental well-being of people and helps them to avoid stress and anxiety [12]. Furthermore, elderly people lose muscle power and cognitive ability with age. Importantly, intervention training such walking, guided tours, museum visitations, and many others are meant to improve their health [13].

The health industry is important for any national economy in several ways. On the one hand, it is highly innovative, research-intensive, and has a high added value, and the increasing human life expectancy is creating a growing demand for improvements in the quality of health and in the products and services provided by the health industry. On the other hand, a country’s economy is significantly affected by the health of its population because individuals cannot work as efficiently when ill; for the employer, the lack of labor leads to a loss in productivity, which increases costs; and for the state/society, it is not a benefit, but an expense [14]. The role of the health sector can be of significance to the economic output both at the individual level and at the country level [15].

However, for health services to work well, healthcare workers need to be physically, mentally, and socially healthy. That is, an adequate quality of healthcare can only be achieved with a highly skilled workforce that is committed to lifelong learning. The shortage of skilled health professionals has serious consequences for the quality, performance, and cost-effectiveness of health services [16], but also limits equal access to health services. According to the WHO data, there is a significant shortage of human resources in this area worldwide, including in Europe, and the situation is projected to worsen [17,18].

The situation is exacerbated by the fact that health indicators for those choosing a healthcare career are strikingly worse than the social average. Several factors contribute to this, one of which is the physical, chemical, biological, and ergonomic risks posed by the healthcare work environment [19]. These include long surgical procedures, patient movements, or office-based sedentary work due to increased administrative workload, but also contamination with biological agents, exposure to chemicals—including disinfectants, anesthetic gases, or antibiotics—and radiation from radiological examinations. A high incidence of accidents at work—including slips, cuts, needle stick injuries, etc.—and aggressive behavior and physical abuse towards healthcare workers are also considered risk factors. They also include stress and fatigue resulting from work schedules. Working hours that are often longer than 8 h per day, working at odd hours, in rotating shifts, and on public holidays also have a negative impact on rest and family relationships. Night work is also a heavy burden. In addition to working hours, the risks are increased by inadequate work organization or high levels of overtime due to staff shortages, and by on-call work outside of normal working hours. Scientific evidence shows that shift work, multi-shift work, and long hours over long periods of time increase the risk of health and social problems [20] and can lead to sleep disorders, cardiovascular and metabolic diseases, and depression.

In addition to the above, a high level of empathy is one of the personality traits of people working in the human sector, as it is the reason why they choose this profession. Their aim is to help people in distress, which is why they work in emotionally charged situations. Furthermore, a significant proportion of them can only earn the income needed for their daily lives by working overtime on a large scale. Overtime, overwork, excessive emotional involvement, and the pressure to perform the job right lead to burnout [21], which can manifest itself in physical, emotional, and/or mental exhaustion [22]. Moreover, working in a healthcare institution and treating and caring for sick people is a moral and legal responsibility and a considerable emotional burden for the healthcare staff. All of these factors play a role in the higher mortality and burnout rates among people working in this field [23,24,25].

Considering the circadian rhythm, one is expected to sleep enough hours at least on a 24 h sleep cycle. Failure to sleep enough can cause many psychological and physical disruptions to occur. Many nursing staffs are faced with this disorder [26]. As the number of skilled nurses or other healthcare workers becomes lower in the labor market as they seek for a new career path with less stress and better working conditions, more and more pressure is added to the available healthcare workers [27]. The negative consequences of night shift work that nurses experience include unhealthy eating habits, increased snacking, a lack of time to practice physical activities, more inactivity, weight gain, and an increased BMI [28]. In addition, the poor income status of the healthcare workers and their high commitments to their jobs lead to high perceived stress, fatigue, poor sleep, and depression [29].

When workers are constantly challenged in their workplace with expectations that are constantly overwhelming in relation to their resources, burnout, fatigue, depression, and health problems occur. One of the best ways to deal with this is to exercise, which helps to relieve tension in the muscles and releases hormones that improve mood, making people feel more energetic and cheerful [30,31]. As described above and in line with previous epidemiological studies, in a sample of US healthcare workers, more leisure-time physical activity was associated with lower feelings of fatigue [32]. Similarly, a Swedish study found that levels of physical activity were inversely correlated with levels of depression, anxiety, and burnout [33]. Spanish researchers studying emergency department workers found that the prevalence of high burnout among workers was nearly 10%, and a lack of physical activity was one of the main predictors of the burnout [34]. US researchers, as a result of a questionnaire survey distributed to nurses, described an increased risk of physical inactivity and obesity in nurses working in non-direct patient care. On the other hand, healthcare workers who find joy in their work experience less stress and have more energy to exercise and prepare healthy meals [35]. German researchers studied the physical activity of shift workers in healthcare institutions and found that the total physical activity performed was not influenced by work schedules. The lower physical activity of shift workers during working hours was compensated by more physical activity outside of working hours [36].

Considering that no similar research was conducted among healthcare workers in Hungary, our investigation can be regarded as a gap-filling study. In light of the above, we were interested to find out how often the employees of one of Hungary’s largest healthcare institutions engage in physical activity or sports and what their lifestyle is. We hypothesized that a smaller percentage of workers in the health sector, typical of the Hungarian population, participate regularly in sports. We also anticipated that female workers are even less likely to participate in sports. We assumed that these are due to fatigue and a lack of leisure time. To verify these hypotheses, a questionnaire survey was carried out.

## 2. Materials and Methods

The survey was carried out among the healthcare workers of the Clinical Center, University of Debrecen, Hungary. The questionnaires were filled in with the help of interviewers and based on self-declaration. The participants’ anonymity was assured. While designing the questionnaire, we took into account international questionnaires on physical activity (IPAQ), but we used a questionnaire that we previously designed with this in mind and which we used and validated in earlier studies [37,38].

In the first part of the questionnaire, we asked for socio-demographic data (7 questions) and leisure time spent during weekdays and on weekends. This was followed by pre-defined multiple choice questions (13) on sporting habits, including how often, what, where, and with whom the respondent preferred to participate in sports, and what motivated them to be physically active. A question was asked about the reasons for not participating in sports; here, several answers could be ticked. The last part of the questionnaire (7 questions) asked about complaints and how often they were experienced, such as backache, headache, stomach ache, nervousness, sleep problems. In this case, a 5-point scale was used. Given that the questionnaire did not contain any personal data, and that the survey was voluntary and anonymous, no ethical clearance was required.

The completed questionnaires were processed using the EvaSys 8.2 software (VSL Inc., Szentendre, Hungary; http://www.vsl.hu). The analysis also examined responses by gender, age group, and marital status, as well as by job and work schedule. The present study involved all employees of the Medical Center, from whom 987 employees responded by filling in the questionnaire, which represents 31% of the total workforce. In the survey, employees were subdivided into the following three job categories: doctors, professional staff (those who have health-related degrees such as nurses, paramedics, medical assistants, etc.), and other workers (all others).

SPSS (Statistical Package for the Social Sciences) 29.0 software (SPSS Inc., Chicago, IL, USA) was used for statistical analysis. The test of normality (Kolmogorov–Smirnov test) showed that the data were not normally distributed; therefore, a non-parametric test, the Kruskal–Wallis test, was used to compare the subgroups. In the case of multiple responses, frequency was analyzed using Multiple Response Frequencies and the correlation between groups was examined using Multiple Response Crosstabs. Pearson’s chi-square test was used for comparing proportions. A *p* value < 0.05 was considered statistically significant.

## 3. Results

The University of Debrecen Clinical Centre employed 3100 healthcare workers at the time of the study. More than 660 doctors, 2000 professional staff, and 400 other workers provide care to nearly 100,000 inpatients and 1,000,000 outpatients every year. In 2018, around 480,000 nursing days and 300,000 specialist doctor hours were completed. The employees are responsible for the healthcare of more than 1.2 million people because of the institution’s territorial coverage obligation. The work and daily commitment of the staff are essential for safe patient care.

**Figure 1:**
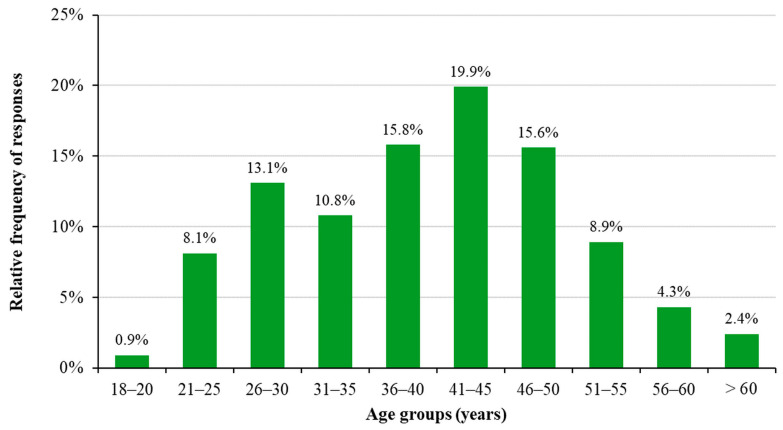
Age distribution of the responding healthcare workers.

The questionnaire was completed by 987 people (approx. 1% of all healthcare workers employed by hospitals in Hungary), with an average age of 40.1 ± 1.5 years (mean ± SD). Of the participants, 16.2% work as doctors, 65.5% work as professional staff, and 18.2% work in other jobs. When analyzing the data by occupational group, the average age of the professional staff is 40.5 ± 1.9 years, that of doctors is 37.5 ± 3.9 years, and that of other professionals is 41.0 ± 3.6 years. The age distribution is shown in Figure 1.

A total of 78.4% (774 people) of the respondents were women, and 21.6% (213 people) were men. The proportion of female and male respondents is almost equal for doctors (46.8–53.2%), while for professional staff and other workers, a female dominance was observed (87.7–12.3%; 66.7–33.3%). In terms of family status, more than half of the respondents (51–55%) in all occupational groups were married. In the other half of the respondents, most were still unmarried, which is particularly significant for doctors (40.3%).

### 3.1. Socio-Economic Factors Influencing Sports Behavior

**Figure 2:**
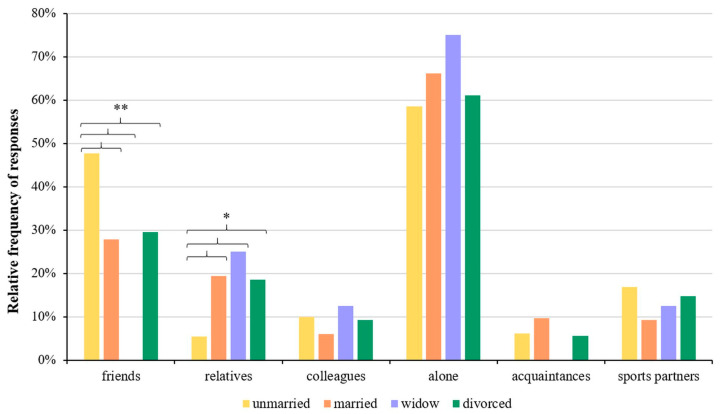
Association with the family status and with whom sports are played. Significant differences (* *p* < 0.05, ** *p* < 0.001; chi-square test) are indicated by asterisks. Respondents were allowed to choose more than one answer.

The relationship between the participants’ family status and with whom they like to participate in sports with was also examined, and we found that on average, 65.2% of the respondents engage in sports alone, 26.3% engage in sports with friends, and 17.1% engage in sports with relatives. There was a significant difference between the groups engaging in sports with friends (47.7%, 27.8%, 0%, and 29.6%; unmarried, married, widowed, and divorced, respectively; *p* < 0.001) and with relatives (5.4%, 19.4%, 25%, and 18.5%, respectively; *p* = 0.011) (Figure 2). The result of the cross-tabulation analysis between the groups engaging in sports with friends and relatives and family status are given in Supplementary Table S1.

We studied the place of residence and observed that 90% of the doctors are from Debrecen, while the other two occupational groups have a higher proportion (33–37%) of travelers from other localities, which can increase fatigue, too. Among the respondents, 37.8% work in 8 h shifts, 39.1% work in 12 h shifts, and 23.1% work in a duty shift schedule.

The respondents estimated that they have an average of 4.0 ± 1.9 h of free time during the week and 5.5 ± 2.3 h on weekends, which is time for leisure to relax and refresh. Females reported significantly less free time than males, both on weekdays (3.9 ± 1.8 vs. 4.3 ± 2.2 h; *p* < 0.02) and weekends (5.3 ± 2.2 vs. 6.0 ± 2.4 h; *p* < 0.001).

**Figure 3:**
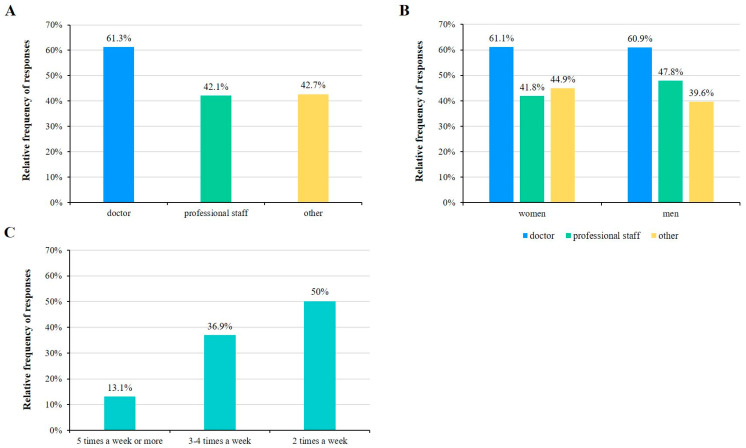
Frequency of participation in sports. The relative proportion of those who participate in sports by occupation (**A**) and by gender (**B**). The relative proportion of how often respondents participate in sports (**C**).

According to the survey, 45.1% of the participants said that they exercise regularly (at least 30 min of continuous physical activity, at least two times per week). Among the respondents, 38.7% of doctors, 57.9% of professional staff, and 57.3% of those working in other jobs do not carry out regular physical activity (Figure 3A). With respect to gender, 56.1% of females and 48.9% of males do not exercise regularly (Figure 3B). Regarding the frequency of sporting activities, the results show that 50% of respondents regularly exercise 2 times per week, while 36.9% answered 3–4 times per week (Figure 3C).

**Figure 4:**
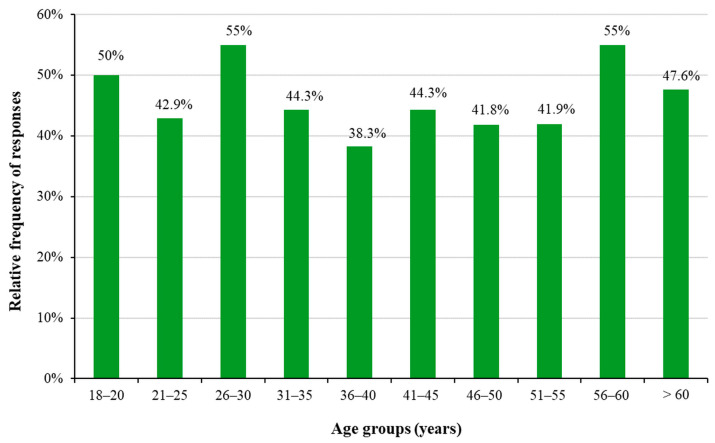
Relative frequency of those engaging in regular physical activity in the different age groups.

When analyzing the relationship between physical activity and age, interestingly, no age dependence was observed (Figure 4). This implies that once someone decides to engage in sports, it prevails, but on the other hand, hardly anybody starts engaging in sports at a later age. This points to the responsibility of the employer to, on the one hand, conduct enlightening trainings to convince their employees to become engaged in sports, and on the other hand, to organize opportunities that give the workers possibilities to engage in sports.

### 3.2. Preferred Places and Types of Sports

**Figure 5:**
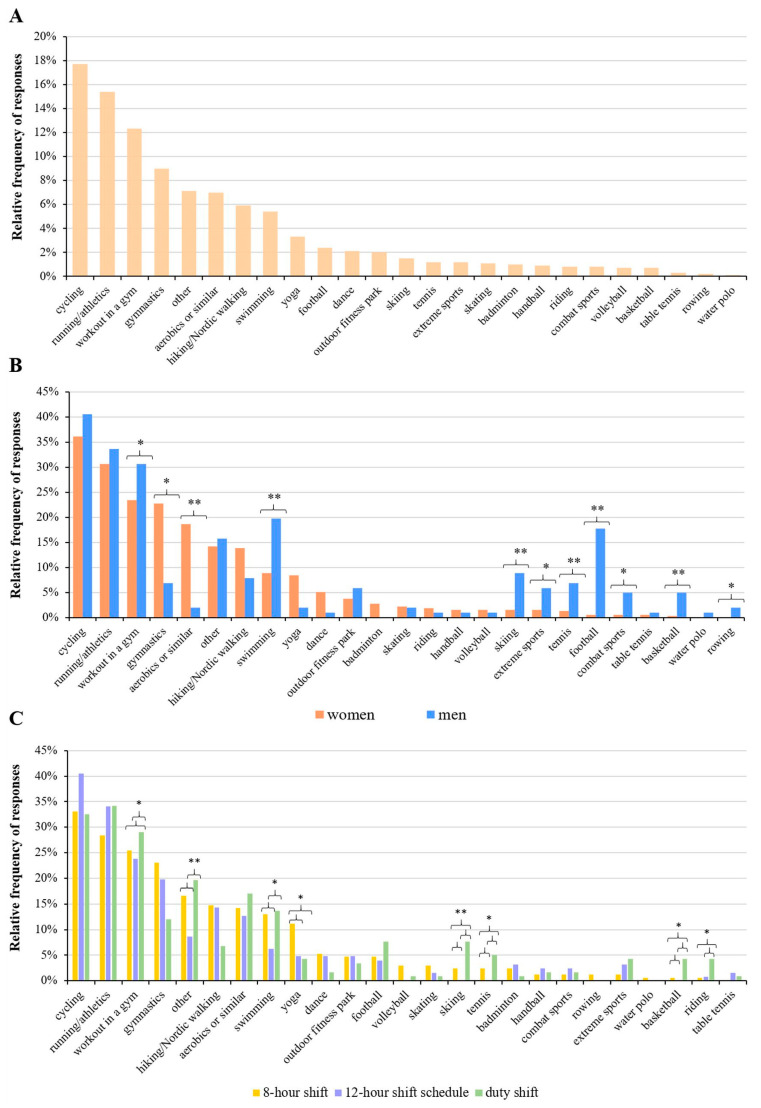
Relative frequency of types of sports played. Data are presented for all respondents (**A**), for the different genders (**B**), and for the different work schedules of the employees (**C**). For the latter two, significant differences (* *p* < 0.05, ** *p* < 0.001; chi-square test) are indicated by asterisks. Respondents were allowed to choose more than one answer.

In terms of the different types of sports, the most common activities are cycling (17.7%), running/athletics (15.4%), and working out in a gym (12.3%) (Figure 5A). When comparing women’s and men’s exercise habits, we observed that women were significantly more likely to choose gymnastics (22.8% and 6.9% of women and men, respectively; *p* = 0.004) and aerobic training (18.7% and 2% of women and men, respectively; *p* < 0.001) than men. Significantly higher percentages of men choose working out in a gym (23.4% and 30.7% of women and men, respectively; *p* = 0.037), swimming (8.9% and 19.8% of women and men, respectively; *p* < 0.001), skiing (1.6% and 8.9% of women and men, respectively; *p* < 0.001), extreme sports (1.6% and 5.9% of women and men, respectively; *p* = 0.008), tennis (1.3% and 6.9% of women and men, respectively; *p* < 0.001), football (0.6% and 17.8% of women and men, respectively; *p* < 0.001), combat sports (0.6% and 5% of women and men, respectively; *p* = 0.001), basketball (0.3% and 5% of women and men, respectively; *p* < 0.001) and rowing (0% and 2% of women and men, respectively; *p* = 0.007) than women (Figure 5B).

When analyzing the types of sport compared with the respondents’ work schedules, we found that working out in a gym (29.1% of responses, *p* = 0.019), swimming (13.7% of responses, *p* = 0.007), skiing (7.7% of responses, *p* < 0.001), tennis (5.1% of responses, *p* = 0.007), basketball (4.3% of responses, *p* = 0.002), riding (4.3% of responses, *p* = 0.01), and other sports (19.7% of responses, *p* < 0.001) were significantly more frequently selected by duty shift groups. Among those working in 8 h shifts, yoga (11.2% of responses, *p* = 0.012) was significantly more commonly chosen (Figure 5C). The results of the cross-tabulation analysis between sport types and work schedules are given in Supplementary Table S2.

Regarding the type of sports played and the occupation, doctors were observed to prefer running, working out in a gym, and cycling, in this order; among professional staff and other workers, cycling is the top activity. Swimming is the fourth most chosen sport by doctors, while professional staff and other workers prefer gymnastics.

**Figure 6:**
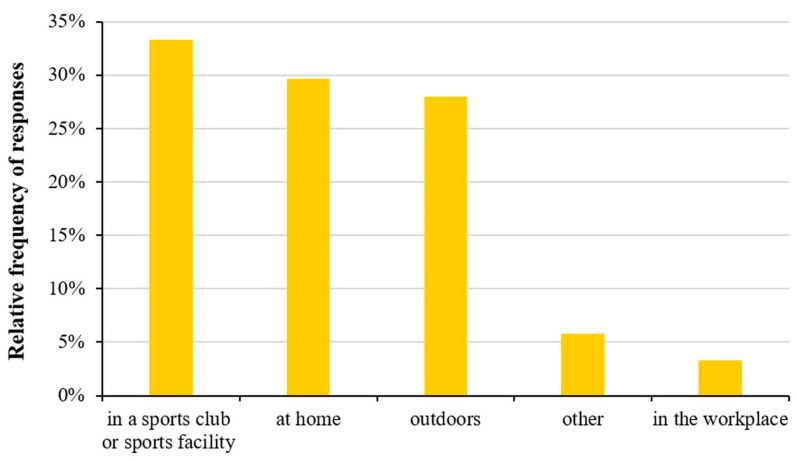
Relative frequency analyses of place for sports.

As for the place where sports are played, the majority of respondents (33.3%) play their chosen sports at a sports club or some kind of sports facility (Figure 6).

Nearly half (44.2%) of the doctors most often choose a sports club or sports facility to exercise, while professional staff and other workers prefer their home and/or outdoor opportunities. We also examined the relationship between satisfaction with the number and quality of sports facilities and the number of sports played at the place of residence. We found that respondents living in a village were the most dissatisfied, while respondents living in a town were the most satisfied with the number and quality of sports facilities and the number of sports that could be played there. However, no statistically significant correlation was detected (Table 1).

Among the respondents, 10.1% are members of a sports club, and 12.8% participate in competitions.

### 3.3. Aspects of Motivation for Engaging or Not Engaging in Physical Activity

**Figure 7:**
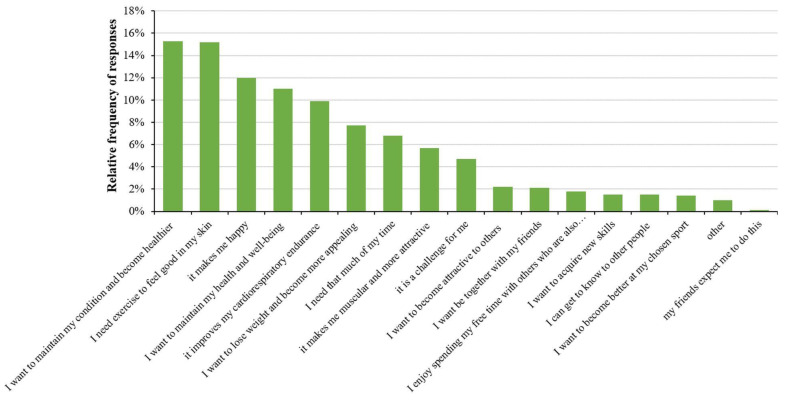
Relative frequency of certain motivation factors for playing sports. Respondents were allowed to choose more than one answer.

The respondents were also asked to evaluate some of the factors that motivate them to engage in sports. According to our results, the most important factors were to maintain their condition and become healthier (15.3% of answers), to feel good in their skin (15.2% of answers), and because they feel happy during exercise (12% of answers) (Figure 7).

**Figure 8:**
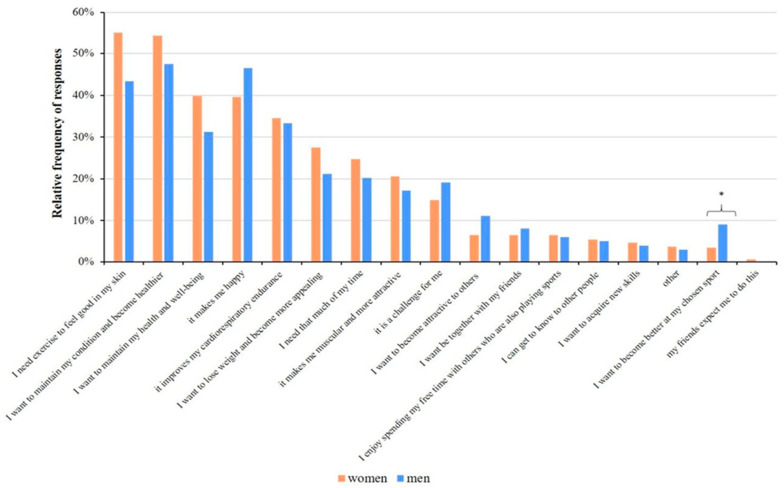
Association with gender and motivation for playing sports. Significant differences (* *p* < 0.05; chi-square test) are indicated by asterisks. Respondents were allowed to choose more than one answer.

An analysis of the gender of the respondents and their motivations showed similar results. Nevertheless, more women responded “I need exercise to feel good in my skin” (55.1% of answers), and men are more entertained and happier (46.5% of answers) by exercising. The only statistically significant difference between women and men for the motivation was seen in the response “I want to become better at my chosen sport” (3.5% and 9.1% of answers by women and men, respectively; *p* = 0.01), as this factor motivated men more than women (Figure 8).

**Figure 9:**
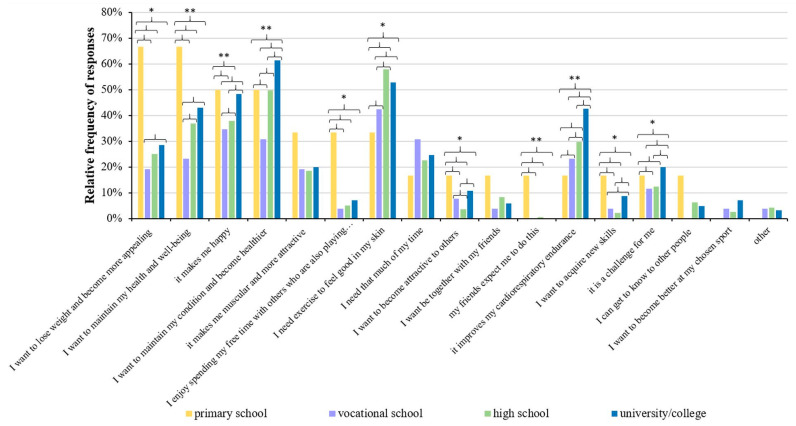
Association with educational attainment and motivation for playing sports. Significant differences (* *p* < 0.05, ** *p* < 0.001; chi-square test) are indicated by asterisks. Respondents were allowed to choose more than one answer.

We also studied the relationship between the educational levels of the respondents and their motivations and found that the majority (66.7%) of participants with elementary school education (1.8% of all respondents) were motivated by the goals of “losing weight to look better” (66.7% of answers) and “maintain my health and well-being” (66.7% of answers). The main motivation for respondents with vocational and high school education (8.9% and 52.6% of all respondents, respectively) was “I need exercise to feel good in my skin” (57.9% and 42.3% of them, respectively). The results show that 61.3% of those with a university or college degree (36.7% of all respondents) play sports because they want to stay fit and become healthier (54.70% of responses). Figure 9 presents the significant differences, marked with asterisks, between the different groups. Regarding the relationship between the motivations of respondents and their occupations, most of the answers were “to maintain my condition and become healthier” and “I need exercise to feel good in my skin” (52.40% of responses) for all types of jobs. The results of the cross-tabulation analysis between the educational level and motivation for playing sports are given in Supplementary Table S3.

**Figure 10:**
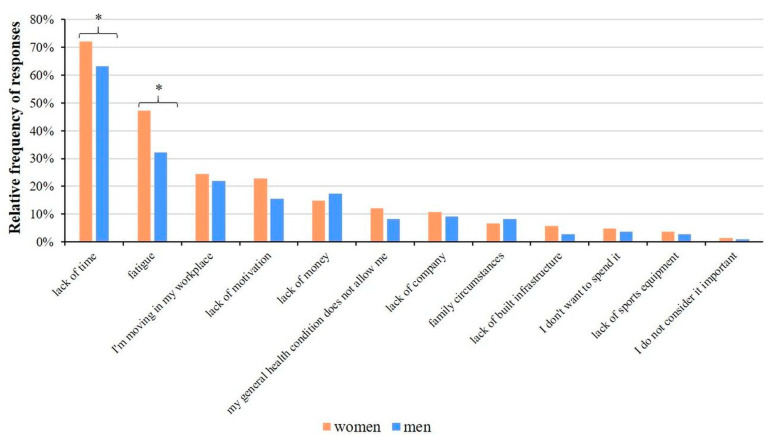
Relative frequency of reasons behind not playing any sports. Significant differences (* *p* < 0.05; chi-square test) are indicated by asterisks. Respondents were allowed to choose more than one answer.

For all job categories, a lack of time and fatigue (on average, 72.67% and 40.8% of responses, respectively) are the main factors which influence physical inactivity. We analyzed the causes of physical inactivity separately for gender and found significant differences for lack of time (72.10% and 63.30% of women and men, respectively; *p* = 0.034) and fatigue (47.20% and 32.10% of women and men, respectively; *p* = 0.003) (Figure 10).

### 3.4. Physical Symptoms of Fatigue and Burnout

We also asked questions about health and well-being in the questionnaire. The data show that employees often experience fatigue (54.3%), back pain (58.7%), and sleeping problems (40%). The frequency of fatigue is significantly higher among women (*p* < 0.001), while back pain is more likely to occur among professional staff (*p* < 0.01) than among doctors. By examining the association between physical activity and health problems, we observed that headaches due to nervousness (*p* = 0.026); back or lower back pain (*p* = 0.015); feeling exhausted, weak, or lethargic (*p* < 0.001); and having a fast or irregular heartbeat (*p* = 0.009) were significantly less common in respondents who engaged in some exercise.

Taken together, the results of our study show that whether someone engages in physical activity regularly depends on their gender, job, and work schedule. The choice of sports is influenced by gender and work schedule, while with whom the respondent prefers to participate in sports is influenced by marital status. The amount of free time, the motivating factor for taking part in sports, and the reasons for not playing sports are influenced by the gender of the respondent.

## 4. Discussion

In our research, we conducted a survey among the staff of one of the largest healthcare institutions in Hungary on their physical activity habits. The results are similar to the previous data obtained among the population in the same region [37,39] and to those of the Special Eurobarometer 472 [40] survey for Hungary, where 46% of the surveyed employees said that they do not exercise. Our results are also consistent with those reported by Saridi and colleagues [41] for Greece, where they described that albeit the physical activity of healthcare workers is low, it, nevertheless, is similar to the overall population in the country. In their study, the lack of physical activity was explained by the respondents to mainly be a consequence of their work schedules and a lack of free time. We also found that the most important reasons for not playing sports were “the lack of time” and “fatigue”. Healthcare workers have a lot of overtime and are overworked, so they have less free time; therefore, we assumed that they play sports less. At the same time, it is known that their task is to draw the attention of patients to the importance of maintaining health and a healthy lifestyle; thus, they are aware that in order to protect their own health, recreation is also important for them, one of the appropriate forms of which is physical activity. Our results show that a lack of time and fatigue override conscious health prevention, even in this special social group.

This is congruent with the fact that health professionals, including doctors, paramedics, and other professionals, are at a higher risk of health problems, including burnout, due to their increased workload (e.g., [42]). Their health can deteriorate faster than that of the average person as the years spent working progresses, which can adversely affect their ability to work. This makes health-conscious measures in the workplace even more justified and necessary for healthcare workers. Since they are pro-health, one would assume that, being aware of its importance, the health behavior and associated activities should be improved and more sustained in this field as compared to the general population. However, this turns out to be false. Their work schedules, as found this work and reported earlier, deprive them of more active participation in physical activity [43]. In comparison between day- and night-shift healthcare workers, it was detected (Figure 5 and related text) that night shift workers experienced more sedentary living than the day shift workers, but in general, their sporting habits were not any better [44]. It is believed that workplace lifestyle interventions provided by employers such as free membership for health fitness clubs targeted to the nurses could help to achieve the desired sporting habits of the healthcare workers for better healthy living and continuous job discharge. Encouraging the workers to dedicate even the smallest amount of time to physical activity would go a long way in positively impacting their health; therefore, work intervention schemes are very crucial to achieving better results [45,46,47].

A human resource function in every organization, be it private or public in any sector or industry in the economy, serves as the backbone or engine to the continuous survival of its operation. Therefore, its significance can never be over-emphasized. No organization, either profit oriented or non-profit oriented, can survive without humans, materials, and financial resources. Therefore, efficient and effective planning is the key to the success of human resource management. In human resource management, employee retention is very crucial to the wider image of an organization. Many experienced employees who are working in another organization but intending to switch to another consider their potential organization’s retention strategy for its existing employees before making an exit decision. This situation in the health sector could be more complicated as it is evident that there is an inadequate number of healthcare workers, and the demand for their services is almost certainly higher. Healthcare employers are in a critical situation to ensure that their employees’ needs are met at all costs and to design programs, incentives, and benefits that are capable of motivating them to stay.

Promoting a healthy lifestyle in place of work is beneficial to not only the staff, but to the organization in general. There are so many associated benefits of encouraging employees to cultivate these habits. The benefits to the employees are very visible as they include improving their health status and active living and improving their wellbeing, as well as many other benefits. Encouraging wellness programs and other health behaviors are crucial to the staff, while to the organization, it is a cost-saving mechanism and enhances productivity [48].

When interpreting the results presented in this report, one should also consider the limitations of this study. Our survey was conducted in a single, albeit the largest healthcare facility in Hungary, and even then, only 30% of the workers filled out the questionnaire. The questionnaire contained predefined options, and there was no possibility to enter free text. One can also assume that if interviews or focus group discussions took place instead of questionnaires, it would have been possible to obtain more in-depth information.

Although, as mentioned above, the survey was conducted among the workers of the largest healthcare institution in Hungary, in order to extrapolate the results of the survey to the entire healthcare workforce in Hungary, several aspects should be taken into account. It is necessary to examine the distribution of healthcare providers within the country, their location in the capital, and whether they are in a regional center or in a small town. In addition to the size of the institutions, the size of the covered area and the epidemiological situation also have an impact on the workload of health workers. These factors not only have a major impact on work-related stress and burnout, but they also have a significant impact on the physical activity of workers outside of working hours. Smaller municipalities typically have less available sports infrastructures (swimming pools, sports halls, ice rinks, etc.) and therefore, fewer choices of sports. It also takes longer to travel to a sports facility, which is likely to reduce the number of people participating in such sports (swimming, ball games, etc.). At the same time, in rural areas, more people are engaged in physical activities other than sports (e.g., cycling, walking, gardening, etc.). For workers commuting in larger cities or from distant places of residence, the time spent travelling increases, which has a significant impact on the amount of leisure time, and therefore, on the amount of time spent playing sports. The location of the study is a county seat and, as such, has an extensive sports infrastructure, but also provides employment opportunities for many commuting workers. The respondents of our survey included a significant number of people from both urban and rural areas, so our survey results can be considered to be close to the national average. In addition, the majority of healthcare workers both in our institution and nationally are women, who typically have less free time than men. This further supports that the results obtained here would be similar to those of a study involving several institutions.

## 5. Conclusions

The fatigue and other negative effects of working at a healthcare institution can lead to many health problems and burnout, and finally, to changes of work. These problems can be mitigated at the individual level by engaging in regular sporting activities and healthy eating, but it would also be necessary for the workplace to provide opportunities for engaging in sports, a healthy menu in the canteen, counselling on healthy lifestyles, screening tests, and organized recreational programs. Health protection at work should be a part of social responsibility, risk assessment, and quality management.

It can be concluded that, in our case, too, there is a need to continue to look for solutions to make regular physical activity accessible and part of the lifestyle of a wider section of society.

## Figures and Tables

**Table 1:** The results of an analysis of the relationship between satisfaction with the number and quality of sports facilities and the number of sports activities in the place of residence.

Place of Residence	Number of Sports Facilities	Quality of Sports Facilities	Number of Sports
	Mean ± Standard deviation		
Debrecen	3.63 ± 1.25	3.67 ± 1.12	3.60 ± 1.19
Another town	3.73 ± 1.03	3.77 ± 0.89	3.54 ± 1.13
Village	3.19 ± 1.39	3.25 ± 1.36	3.33 ± 1.41
Farm ^b^	1.00 ± 0.0	1.00 ± 0.0	1.00 ± 0.0
H (df = 3)	6.48	6.24	3.95
*p*-value ^a^	0.091	0.100	0.267

^a^ Kruskal–Wallis test. ^b^ All respondents answered with 1 as there are no such facilities in a farm.

## Data Availability

The datasets generated and analyzed during this study are available from the corresponding author upon reasonable request.

## Supplementary Materials

The following supporting information can be downloaded at https://www.mdpi.com/article/10.3390/healthcare11131915/s1, Table S1: The result of the cross-tabulation analysis between the groups engaging in sports with friends and relatives and family status, Table S2: The result of the cross-tabulation analysis between types of sports played and work schedule, Table S3: The result of the cross-tabulation analysis between the educational attainment and motivation for playing sports.
