# Supplementary material for: The Importance of Physical Activity in Preventing Fatigue and Burnout in Healthcare Workers
Source: Healthcare (Basel). 2023 Jul 3;11(13):1915. doi: 10.3390/healthcare11131915 (PMC10340398; doi:10.3390/healthcare11131915)
Supplement: Supplementary file 1 [file healthcare-11-01915-s001.zip › Supplementary Table S1.pdf]

Supplementary Table S1. The result of the cross-tabulation analysis between the groups sporting with friends and relatives and family status.

**Sporting with FRIENDS**	**Family status**			
	Unmarried	Married	Widow	Divorced
Unmarried		p<0.001	p<0.001	p<0.001
Married	p<0.001		p<0.001	p>0.05
Widow	p<0.001	p<0.001		p<0.001
Divorced	p<0.001	p>0.05	p<0.001	

**Sporting with RELATIVES**	**Family status**			
	Unmarried	Married	Widow	Divorced
Unmarried		p<0.05	p<0.05	p<0.05
Married	p<0.05		p>0.05	p>0.05
Widow	p<0.05	p>0.05		p>0.05
Divorced	p<0.05	p>0.05	p>0.05	
