# Supplementary material for: The Importance of Physical Activity in Preventing Fatigue and Burnout in Healthcare Workers
Source: Healthcare (Basel). 2023 Jul 3;11(13):1915. doi: 10.3390/healthcare11131915 (PMC10340398; doi:10.3390/healthcare11131915)
Supplement: Supplementary file 1 [file healthcare-11-01915-s001.zip › Supplementary Table S2.pdf]

Supplementary Table S2. The result of the cross-tabulation analysis between types of sports played and work schedule.

**Workout in a gym**	**Work schedule**		
	8-hour shift	12-hour shift	Duty shift
8-hour shift		$p>0.05$	$p<0.05$
12-hour shift	$p>0.05$		$p<0.05$
Duty shift	$p<0.05$	$p<0.05$	

**Other**	**Work schedule**		
	8-hour shift	12-hour shift	Duty shift
8-hour shift		$p<0.001$	$p>0.05$
12-hour shift	$p<0.001$		$p<0.001$
Duty shift	$p>0.05$	$p<0.001$	

**Swimming**	**Work schedule**		
	8-hour shift	12-hour shift	Duty shift
8-hour shift		$p<0.05$	$p>0.05$
12-hour shift	$p<0.05$		$p<0.05$
Duty shift	$p>0.05$	$p<0.05$	

**Yoga**	**Work schedule**		
	8-hour shift	12-hour shift	Duty shift
8-hour shift		$p<0.05$	$p<0.05$
12-hour shift	$p<0.05$		$p>0.05$
Duty shift	$p<0.05$	$p>0.05$	

**Skiing**	**Work schedule**		
	8-hour shift	12-hour shift	Duty shift
8-hour shift		$p<0.001$	$p<0.001$
12-hour shift	$p<0.001$		$p<0.001$
Duty shift	$p<0.001$	$p<0.001$	

**Tennis**	**Work schedule**		
	8-hour shift	12-hour shift	Duty shift
8-hour shift		$p<0.05$	$p<0.05$
12-hour shift	$p<0.05$		$p<0.05$
Duty shift	$p<0.05$	$p<0.05$	

**Basketball**	**Work schedule**		
	8-hour shift	12-hour shift	Duty shift
8-hour shift		$p<0.05$	$p<0.05$
12-hour shift	$p<0.05$		$p<0.05$
Duty shift	$p<0.05$	$p<0.05$	

**Riding**	**Work schedule**		
	8-hour shift	12-hour shift	Duty shift
8-hour shift		$p>0.05$	$p<0.05$
12-hour shift	$p>0.05$		$p<0.05$
Duty shift	$p<0.05$	$p<0.05$	
