# Supplementary material for: The Importance of Physical Activity in Preventing Fatigue and Burnout in Healthcare Workers
Source: Healthcare (Basel). 2023 Jul 3;11(13):1915. doi: 10.3390/healthcare11131915 (PMC10340398; doi:10.3390/healthcare11131915)
Supplement: Supplementary file 1 [file healthcare-11-01915-s001.zip › Supplementary Table S3.pdf]

Supplementary Table S3. The result of the cross-tabulation analysis between the educational attainment and motivation for doing sports.

**Motivation**	**Educational level**			
**„losing weight to look better”**	Primary school	Vocational school	High school	University/college
Primary school		$p<0.05$	$p<0.05$	$p<0.05$
Vocational school	$p<0.05$		$p>0.05$	$p<0.05$
High school	$p<0.05$	$p>0.05$		$p>0.05$
University/college	$p<0.05$	$p<0.05$	$p>0.05$	

**Motivation**	**Educational level**			
**„maintain my health and well-being”**	Primary school	Vocational school	High school	University/college
Primary school		$p<0.001$	$p<0.001$	$p<0.001$
Vocational school	$p<0.001$		$p<0.001$	$p<0.001$
High school	$p<0.001$	$p<0.001$		$p>0.05$
University/college	$p<0.001$	$p<0.001$	$p>0.05$	

**Motivation**	**Educational level**			
**„it makes me happy”**	Primary school	Vocational school	High school	University/college
Primary school		$p<0.001$	$p<0.001$	$p>0.05$
Vocational school	$p<0.001$		$p<0.001$	$p<0.001$
High school	$p<0.001$	$p<0.001$		$p<0.001$
University/college	$p>0.05$	$p<0.001$	$p<0.001$	

**Motivation**	**Educational level**			
**„to maintain my condition and become healthier”**	Primary school	Vocational school	High school	University/college
Primary school		$p<0.001$	$p>0.05$	$p<0.001$
Vocational school	$p<0.001$		$p<0.001$	$p<0.001$
High school	$p>0.05$	$p<0.001$		$p<0.001$
University/college	$p<0.001$	$p<0.001$	$p<0.001$	

**Motivation**	**Educational level**			
**„enjoy spending my free time with others”**	Primary school	Vocational school	High school	University/college
Primary school		$p<0.05$	$p<0.05$	$p<0.05$
Vocational school	$p<0.05$		$p>0.05$	$p>0.05$
High school	$p<0.05$	$p>0.05$		$p>0.05$
University/college	$p<0.05$	$p>0.05$	$p>0.05$	

**Motivation**	**Educational level**			
**„to feel good in my skin”**	Primary school	Vocational school	High school	University/college
Primary school		$p<0.05$	$p<0.05$	$p<0.05$
Vocational school	$p<0.05$		$p<0.05$	$p<0.05$
High school	$p<0.05$	$p<0.05$		$p>0.05$
University/college	$p<0.05$	$p<0.05$	$p>0.05$	

**Motivation**	**Educational level**			
**„to become attractive to others”**	Primary school	Vocational school	High school	University/college
Primary school		$p<0.05$	$p<0.05$	$p<0.05$
Vocational school	$p<0.05$		$p<0.05$	$p>0.05$
High school	$p<0.05$	$p<0.05$		$p<0.05$
University/college	$p<0.05$	$p>0.05$	$p<0.05$	

**Motivation**	**Educational level**			
**„my friends expect me to do this”**	Primary school	Vocational school	High school	University/college
Primary school		$p<0.001$	$p<0.001$	$p<0.001$
Vocational school	$p<0.001$		$p>0.05$	$p>0.05$
High school	$p<0.001$	$p>0.05$		$p>0.05$
University/college	$p<0.001$	$p>0.05$	$p>0.05$	

**Motivation**	**Educational level**			
**„improves my cardiorespiratory endurance”**	Primary school	Vocational school	High school	University/college
Primary school		$p<0.001$	$p<0.001$	$p<0.001$
Vocational school	$p<0.001$		$p<0.001$	$p<0.001$
High school	$p<0.001$	$p<0.001$		$p<0.001$
University/college	$p<0.001$	$p<0.001$	$p<0.001$	

**Motivation**	**Educational level**			
**„to acquire new skills”**	Primary school	Vocational school	High school	University/college
Primary school		$p<0.05$	$p<0.05$	$p<0.05$
Vocational school	$p<0.05$		$p>0.05$	$p<0.05$
High school	$p<0.05$	$p>0.05$		$p<0.05$
University/college	$p<0.05$	$p<0.05$	$p<0.05$	

**Motivation**	**Educational level**			
**„it is a challenge for me”**	Primary school	Vocational school	High school	University/college
Primary school		$p<0.05$	$p<0.05$	$p<0.05$
Vocational school	$p<0.05$		$p>0.05$	$p<0.05$
High school	$p<0.05$	$p>0.05$		$p<0.05$
University/college	$p<0.05$	$p<0.05$	$p<0.05$	
